# Seasonal and diurnal patterns of soil respiration in an evergreen coniferous forest: Evidence from six years of observation with automatic chambers

**DOI:** 10.1371/journal.pone.0192622

**Published:** 2018-02-12

**Authors:** Naoki Makita, Yoshiko Kosugi, Ayaka Sakabe, Akito Kanazawa, Shinjiro Ohkubo, Makoto Tani

**Affiliations:** 1 Faculty of Science, Shinshu University, Nagano, Japan; 2 Graduate School of Agriculture, Kyoto University, Kyoto, Japan; 3 Public Works Research Institute, Tsukuba, Japan; 4 NARO Hokkaido Agricultural Research Center, Hokkaido, Japan; 5 Department of Environments and Conservation, University of Human Environments, Aichi, Japan; Tennessee State University, UNITED STATES

## Abstract

Soil respiration (*R*_s_) plays a key role in the carbon balance of forest ecosystems. There is growing evidence that *R*_s_ is strongly correlated with canopy photosynthesis; however, how *R*_s_ is linked to aboveground attributes at various phenological stages, on the seasonal and diurnal scale, remains unclear. Using an automated closed dynamic chamber system, we assessed the seasonal and diurnal patterns of *R*_s_ in a temperate evergreen coniferous forest from 2005 to 2010. High-frequency *R*_s_ rates followed seasonal soil temperature patterns but the relationship showed strong hysteresis. Predictions of *R*_s_ based on a temperature-response model underestimated the observed values from June to July and overestimated those from August to September and from January to April. The observed *R*_s_ was higher in early summer than in late summer and autumn despite similar soil temperatures. At a diurnal scale, the *R*_s_ pattern showed a hysteresis loop with the soil temperature trend during the seasons of high biological activity (June to October). In July and August, *R*_s_ declined after the morning peak from 0800 to 1400 h, although soil temperatures continued to increase. During that period, figure-eight-shaped diurnal *R*_s_ patterns were observed, suggesting that a midday decline in root physiological activity may have occurred in early summer. In September and October, *R*_s_ was higher in the morning than in the night despite consistently high soil temperatures. We have characterised the magnitude and pattern of seasonal and diurnal *R*_s_ in an evergreen forest. We conclude that the temporal variability of *R*_s_ at high resolution is more related to seasons across the temperature dependence.

## Introduction

Knowledge of soil carbon (C) dynamics is essential for understanding the C balance in terrestrial ecosystems [1]. Gross primary production (GPP) and soil respiration (*R*_s_) are major CO_2_ fluxes between the atmosphere and terrestrial ecosystems. *R*_s_ accounts for more than two-thirds of ecosystem respiration (98 ± 12 Pg CO_2_ yr^−1^) [2]. Even a small change in the CO_2_ release via *R*_s_ processes would have a significant effect on atmospheric CO_2_ concentration and potentially affect climate change [3,4]. Therefore, *R*_s_ is likely to be an important determinant of ecosystem C balance under future climate change scenarios.

Forest *R*_s_ shows significant temporal variation and is affected by environmental factors that control the metabolism of root- and soil-living organisms. It is also affected by environmental conditions controlling gaseous diffusion and convection [5,6]. Among the environmental factors, soil temperature is the most important abiotic factor controlling *R*_s_ [7]. Over the past decade, automated systems for recording *R*_s_ have been developed, providing temporally dense datasets [8,9]. Manual systems effectively cover spatial variability; however, automated monitoring enables the analysis of temporal variations in *R*_s_ rates during conditions such as nighttime and rainfall when manual measurements are impracticable [9–11]. This high temporal resolution also makes it possible to observe the response of *R*_s_ to rapid temporal changes in environmental conditions effectively without the use of linear interpolation or models [12,13].

As the automated chamber method has developed, there is growing evidence that *R*_s_ is closely correlated with C flux from aboveground to belowground over time scales ranging from hours to days and months [14–16]. Data from automated chambers indicate that *R*_s_ rates correspond to changes in canopy photosynthesis and environmental parameters directly affecting leaf CO_2_ gas exchange, such as photosynthetic photon flux density and vapor pressure deficit [13,14,17]. Consequently, annual variations in the observed *R*_s_ do not always coincide with model estimates based on soil environmental factors [18,19].

On the seasonal scale, it is becoming increasingly evident that temporal variations in forest C balance and C allocation have a strong phenological component [20,21]. Aboveground, leaf phenology is characterized by seasonal patterns of growth and senescence. A recent study highlighted critical feedbacks between variation in leaf phenology and ecosystem productivity [22]. The timing of leaf development in spring and leaf senescence and abscission in autumn indicates the variability in C balance and C allocation in the trees. On the other hand, belowground phenology is characterized by pulses of root production during periods conducive to plant growth [23]. For many species, a primary flush in root production occurs between late spring and summer [24,25]. When root proliferation occurs in the spring, the amount of respiring tissue increases with temperature-dependent CO_2_ effluxes to maintain root and mycorrhizal growth [26–28]. In this case, root respiration should reflect a combination of seasonal root growth variations and temperature responses to specific respiration rates. Nevertheless, less is known about the phenological pattern of *R*_s_, which may be further complicated as patterns change with soil temperature. Quantifying the seasonality of these *R*_s_ processes is useful for improving models of ecosystem productivity and global biogeochemistry [3,4].

Another advantage of the automated system is that it can evaluate diurnal scales. Recent studies using measurements with high temporal resolution have shown that *R*_s_ can vary during the day at a given soil temperature, causing a diurnal hysteresis in the temperature–respiration relationship [29–31]. Phase lags between the diurnal signals of soil temperature and *R*_s_ have been reported [28, 32], resulting from processes such as photosynthate supply, heat transport, and CO_2_ diffusion [33,34]. The supply of substrate to roots and soil microbes is a critical determinant of variations in *R*_s_ [7,15] and accurate annual *R*_s_ budgets [19]. Nevertheless, the diurnal patterns of *R*_s_ rate for each season remain unclear [35]. A recent study showed that C transport rates vary seasonally and are affected by soil environmental conditions [36–38]. Plant phenology potentially affects diurnal rhythms of whole-tree physiology (e.g., assimilate supply) and growth in forest ecosystems, which can influence the semi-elliptical shapes of the *R*_s_-soil temperature regression curves [39]. Therefore, in forests, we suggest that the differences in diurnal patterns of *R*_s_ may be due to seasonal variations.

The present study aimed to characterize seasonal and diurnal patterns of *R*_s_ in a temperate evergreen coniferous forest consisting primarily of *Chamaecyparis obtusa* (Japanese cypress). To this end, *R*_s_ was measured at 30-min intervals for 6 years by an automated closed dynamic chamber system. The present work builds on the study of Kosugi et al. [40], in which CO_2_ gas exchange between the atmosphere and an evergreen coniferous forest was determined using eddy covariance flux data at the same study site as that of the present study. The authors reported that the temperature dependence of canopy photosynthesis decreased significantly in winter and that plant phenology must be considered to understand the seasonality of forest CO_2_ exchange. Nevertheless, few studies have linked *R*_s_ patterns in evergreen forests to seasonal differences in phenology. We tested the hypothesis that *R*_s_ shows clear diurnal and seasonal changes beyond the semi-empirical model of the response of *R*_s_ to soil temperature factors in an evergreen forest. Furthermore, we tested the hypothesis that the diurnal pattern of *R*_s_ would be influenced by seasonality.

## Materials and methods

### Study site

The study was conducted in a temperate coniferous forest in Kiryu Experimental Watershed (35°N, 136°E; 190–255 m above sea level; 5.99 ha) located in Shiga Prefecture, central Japan. The region has a monsoon climate. The forest consists of 50-year-old Japanese cypress (*Chamaecyparis obtusa* Sieb. et Zucc.) planted in 1959. The mean tree height (diameter at breast height [DBH] > 5 cm) was 17.3 m based on the tree census in March 2011. The annual mean air temperature and precipitation between 2005 and 2010 at this site were 13.4°C and 1595 mm yr^−1^, respectively (S1 Fig). This region has a distinct climate; it has cold winters with little snow and hot, humid summers with high rainfall owing to the significant effect of the Asian monsoon. The mean monthly air temperature was the highest in August (25.0°C) and the lowest in January (2.8°C). This area typically has snowfall on several days during a year, which melts within a few days. Rain occurs throughout the year, with two peaks in summer: the early summer *baiu* front season and the late summer typhoon season. Summer in western Japan is warm and humid with sufficient rain; however, occasional moderate drought conditions can occur (S1 Fig). The soil is classified as a Haplic Cambisol with sandy loam or loamy sand texture. The mean C/N ratio, pH, and electrical conductivity of the 0–5 cm mineral soil layer were 19.0, 5.9, and 4.9 mS/m, respectively [41].

The study forest is one of the Asia Flux sites. Micrometeorological and CO_2_/H_2_O flux data were collected by the observation tower [40,42]. To compare the net ecosystem exchange estimated by the eddy covariance method, CO_2_ and H_2_O exchanges of leaves [40], manual soil CO_2_ efflux [43], and soil CH_4_ flux [41] were evaluated at this site. The average and standard deviation of annual GPP, ecosysytem respiration, and net ecosystem exchange were 2044 ± 149, 1555 ± 158, and −490 ± 109 g C m^−2^ yr^−1^, respectively [40].

### Measurement of *R*_s_, soil environment, and GPP

Three measurement plots were established in the study area, separated from each other by ≥ 25 m. *R*_s_ was measured continuously with high temporal resolution at one point per plot at 30-min intervals from 2005 to 2010. Measurements were performed with an automated closed dynamic chamber system fitted with an infrared CO_2_/H_2_O analyzer (Li-840; Li-cor, Lincoln, NE, USA). The system consisted of a permanently connected chamber (length 0.3 m, width 0.3 m, height 0.2 m) with an automatically controlled chamber lid. To minimize error in the CO_2_ efflux measurements in closed dynamic chambers through pressure changes, the chambers were designed to provide sufficient volume for the steady pressure in the closed-chamber. The soil collars were inserted tightly into the ground up to 5 cm in depth prior to the start of the sampling period and were sealed permanently to the chamber. Chamber opening and closing were controlled by an air compressor (FH-02; MEIJI, Japan). Switching between chambers was regulated by the air flow from solenoid valves (CKD USB3-6-3-E; CKD Corp., Japan) and AC/DC controller (SDM-CD16AC; Campbell Scientific, USA). To prevent shadow on the collar, all chamber material was consisted of transparent acrylic. When the chamber was closed, the air sample was dehydrated with a gas dryer to remove water vapor in the sample air and then circulated by a mass flow-controlled diaphragm pump (APN-085; Iwaki Pumps, Japan; DM-403ST-25; MFG. CO., LTD., Japan) through polyethylene tubes to the CO_2_/H_2_O analyzer. The flow rate using a mass flow controller (MPC0005; Yamatake, Japan) was 1.8 L min^−1^. Because not all of the water vapor could be removed by the drying system (PD-50 T-48; Perma Pure, Toms Rivers, NJ, USA), its presence was corrected by using the H_2_O concentration measured with the CO_2_/H_2_O analyzer. The time interval for each measurement was set to 180 s. To compensate for air disturbances caused by opening the chamber, the data for the first 90 s were discarded. Measurements were taken every 30 min. Data were recorded with a data logger (CR1000; Campbell Scientific, USA). The closed chamber flux measurement was accepted if the determination coefficient of linear regression (R^2^) was larger than 0.85 according to the previous reports [11,41].

*R*_s_ was calculated from the rate of increase in CO_2_ concentration with time using the following linear regression:

$$Rs=\frac{dc}{dt}\times \frac{V}{A}\times \rho_{airmol}$$ (Eq 1)

where *dc*/*dt* is the rate of increase in the gas concentration *c* (ppm) with time *t* (s) and is determined by the linear least-squares method on the slope of the change in gas concentration from 90 to180 s at the start of measurement; *V* is the chamber volume (0.018 m^3^); *A* is the soil surface area in the chamber (0.09 m^2^); and *ρ*_*airmol*_ is the air molar density (mol m^−3^).

For soil environmental monitoring, soil temperatures at 2-cm depth were measured using copper-constantan thermocouples. Soil moisture levels at 0–30 cm depth were determined with three water content reflectometers (CS615 or CS616; Campbell Scientific, USA). Data were logged continuously at each plot at 30-min intervals. Precipitation was measured with a tipping-bucket rain gauge at an open screen site near the flux tower.

For evaluating GPP, the fluxes of CO_2_ (μmol m_−2_ s_−1_) were measured by open-path eddy covariance methods at a tower height of 28.5 m with a CO_2_/H_2_O gas analyzer (LI-7500; Li-cor, Inc., Lincoln, NE, USA). from January 2005 to December 2010. The study by Kosugi et al. [40] provides detailed information regarding the eddy covariance flux observations and calculations.

### Soil respiration models

To estimate the best fit of soil temperature control on *R*_s_ rates, two empirical models, i.e., the simple exponential function model and the Arrhenius equation model, were tested. Because of the complexity of the soil environment, many researchers depend on empirical models instead of process-based models to estimate soil respiration [7]. The simplest model is the exponential increase in respiration rate as a function of temperature. The model and its parameter space are defined as

$$Rs={\mathrm{Rs}}_{\mathrm{ref}}\times Q_{10}{}^{\frac{Tsoil-Tref}{10}}$$ (Eq 2; *Q*_10_ model)

where *R*s_ref_ > 0 and a1 > 0. *R*_s_ and *R*s_ref_ are the respiration rates (μmol m^−2^ s^−1^) at temperatures *T*_soil_ and *T*_ref_, respectively. *T*_soil_ is the observed soil temperature and *T*_ref_ = 15°C. *Q*_10_ is the temperature sensitivity and represents the relative increase in respiration as the temperature rises by 10°C. Eq 2 is often called the *Q*_10_ model.

The second model is the Arrhenius equation. It is also used to describe temperature dependence of respiration [44]. Since respiration increases with temperature, this model and its parameter space are defined as

$Rs={\mathrm{Rs}}_{\mathrm{ref}}e^{\;\frac{-E_{a}}{R\times Tsoil}}$ (Eq 3; Arrhenius model)

where *E*_a_ is a free parameter analog to the activation energy in the standard Arrhenius model and represents the sensitivity of *R*_s_ to temperature. R is the gas constant (R = 8.314 J K^−1^ mol^−1^). Eq 3 (the Arrhenius model) can predict the behavior of chemical systems according to enzyme kinetics that describe the relationships between enzyme activity and temperature.

### Data analysis

To remove outliers, residual analyses were performed. Data points of *R*_s_ were removed from the regression when the residual of an individual data point was greater than three times the standard deviation. *R*_s_ was calculated as the mean of the three chambers and was used in subsequent analyses. Instrument failure and quality control procedures reduced the data by 10% during the 6 years of observation. We evaluated the empirical models of soil respiration at each soil temperature for the years from 2005 to 2010. Two commonly used models (Eqs 2 and 3), both of which fit the data well, were used to analyze the response of *R*_s_ to soil temperature. The Akaike information criterion (AIC) and the root mean squared error (RMSE) were used to evaluate the goodness of fit for the *R*_s_ models. The observed *R*_s_ and predicted *R*_s_ by the best-fit *R*_s_-temperature model were calculated to determine the direction and magnitude of the seasonal dependence of *R*_s_ measurements beyond temperature-response property. To better characterize seasonal *Q*_10_ and *E*_a_, monthly mean values were caluculated for the years from 2005 to 2010.

The mean diurnal cycles of *R*_s_ and GPP for each month were determined by calculating the average of the 30-min data at each time of day. The cycles were then used to identify the relationship between *R*_s_ and soil temperature.

## Results

### Soil environmental factors and carbon exchange over six years

**Fig 1:**
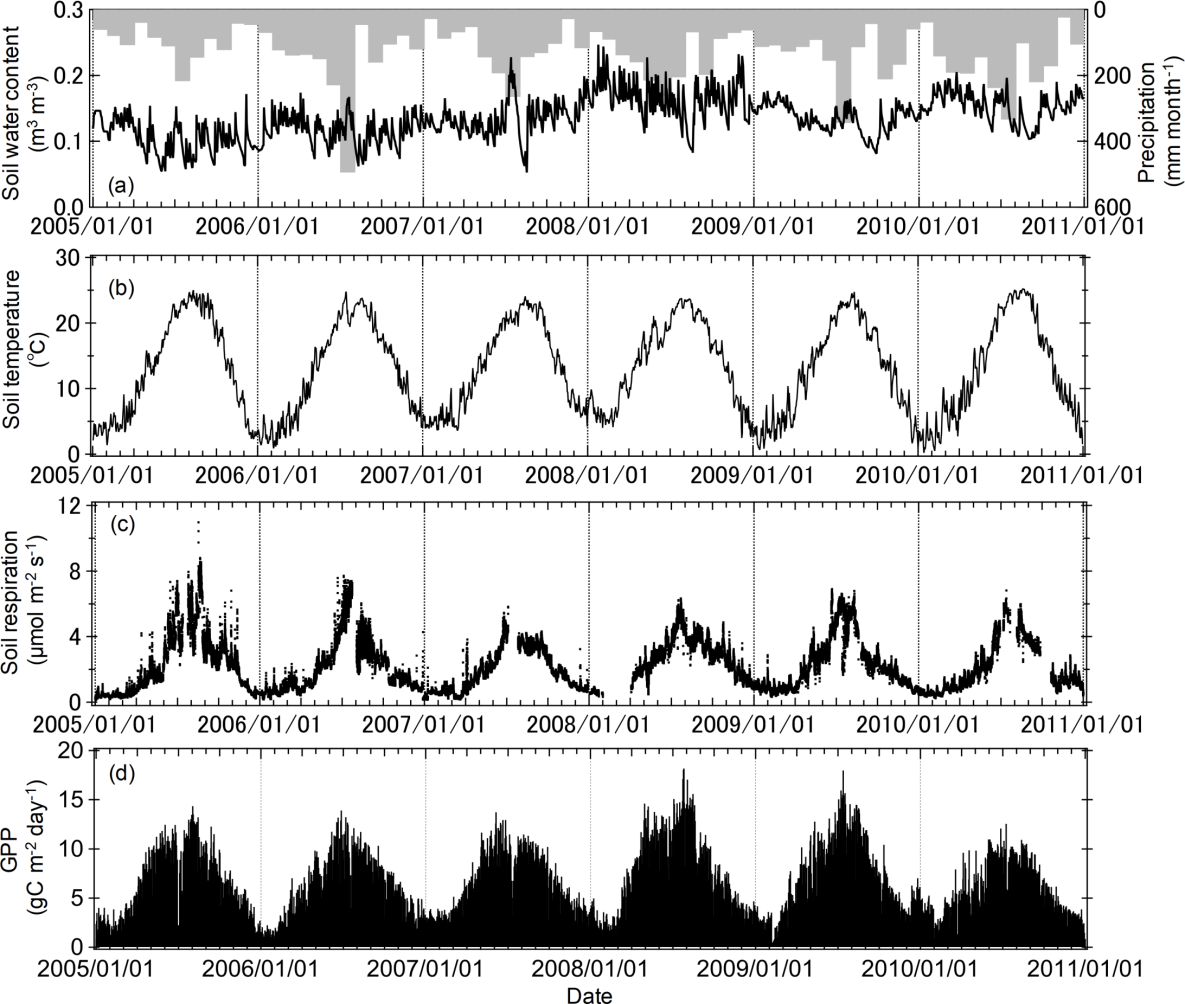
Time courses of (a) mean soil water content at 0–30 cm depth (n = 3) and precipitation levels, (b) mean soil temperature at 2 cm depth (n = 3), (c) half-hourly mean soil respiration rates (n = 3), (d) gross primary production (GPP) according to eddy covariance tower observations during the years from 2005 to 2010.

The mean soil water content at 0–30 cm depth ranged from 0.05 to 0.24 m^3^ m^−3^ of soil (Fig 1A). Seasonal soil temperature patterns were observed (Fig 1B). The mean soil temperature at 2 cm depth varied seasonally, ranging from 0°C in February to 25°C in August during the years from 2005 to 2010. The half-hourly *R*_s_ rates measured with the automated chamber ranged from 0.1 to 10.9 μmol m^−2^ s^−1^ during the years from 2005 to 2010 (Fig 1C). *R*_s_ showed strong seasonality; it was the lowest in February and the highest in mid-August. Seasonal variations in daily GPP over the course of this study are illustrated in Fig 1D.

### Seasonal variation of soil respiration in relation to temperature and gross primary production

Two models of the correlation between *R*_s_ and soil temperature were tested to obtain the best-fit curves. RMSE and AIC based on the *R*_s_-soil temperature relationship were smaller in the Arrhenius model than in the *Q*_10_ model (Table 1). When pooling data of all seasons, the *Q*_10_ and *E*_a_ value was 2.42 and 61.69 kJ mol^−1^, respectively. A better fit for the Arrhenius model was found for the relationship of *R*_s_ with soil temperature for the years from 2005 to 2010 and was used in further analyses.

**Table 1:** Empirical equations and parameter estimates describing the relationship between soil respiration and temperature from 2005 to 2010 (n = 94904). The Akaike information criterion (AIC) and the root mean squared error (RMSE) are used to evaluate the best fit for the models.

Model	Equation and parameter estimates	RMSE	AIC
*Q*_10_ model	$Rs={\mathrm{Rs}}_{\mathrm{ref}}\times Q_{10}{}^{\frac{Tsoil-Tref}{10}}=0.57\times 2.42^{\frac{Tsoil-15}{10}}$	0.68	196173
Arrhenius model	$Rs={\mathrm{Rs}}_{\mathrm{ref}}e^{\frac{‑\mathrm{Ea}}{R\times Tsoil}}=2.18e^{\;\frac{‑61692}{8.31\times \mathrm{Tsoil}}}$	0.67	194786

**Fig 2:**
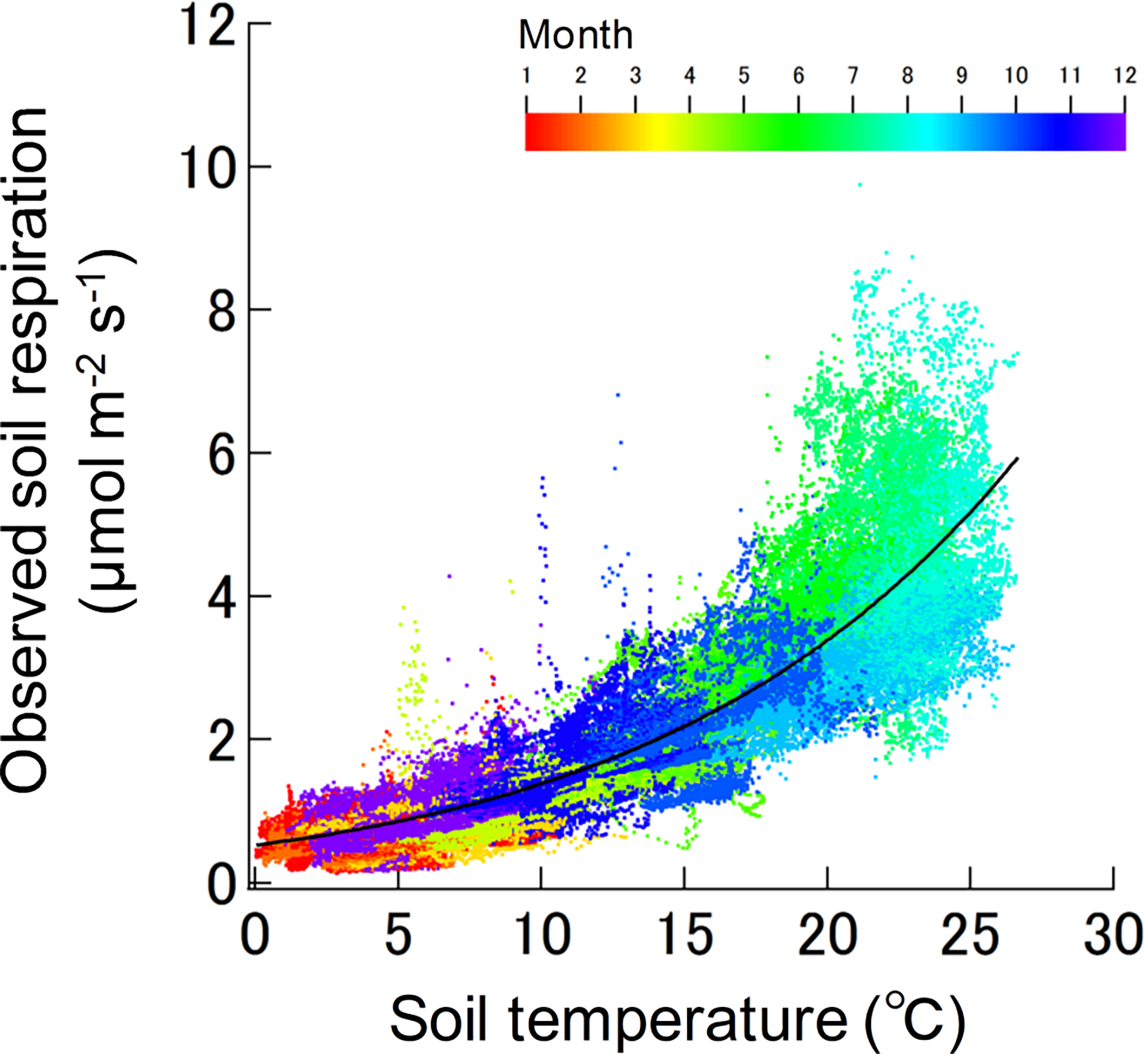
Relationship between soil respiration and temperature during 2005–2010 as determined by the automated chamber system. The best-fit linear relationship from the Arrhenius model is shown by the solid black line (Table 1). The rainbow color scale shows the month when the data were obtained.

In all seasons, *R*_s_ exponentially increased with soil temperature (Fig 2). The Arrhenius model explained a significant portion of the variation in *R*_s_ in response to soil temperature (Table 1). Monthly mean values of observed *R*_s_ were the highest in July and the lowest in February. In contrast, the monthly predicted *R*_s_ were the highest in August and the lowest in February. The underestimations of the predicted- to observed *R*_s_ were found for June-July. In contrast, the overestimations were observed for January−May and August-September.

**Fig 3:**
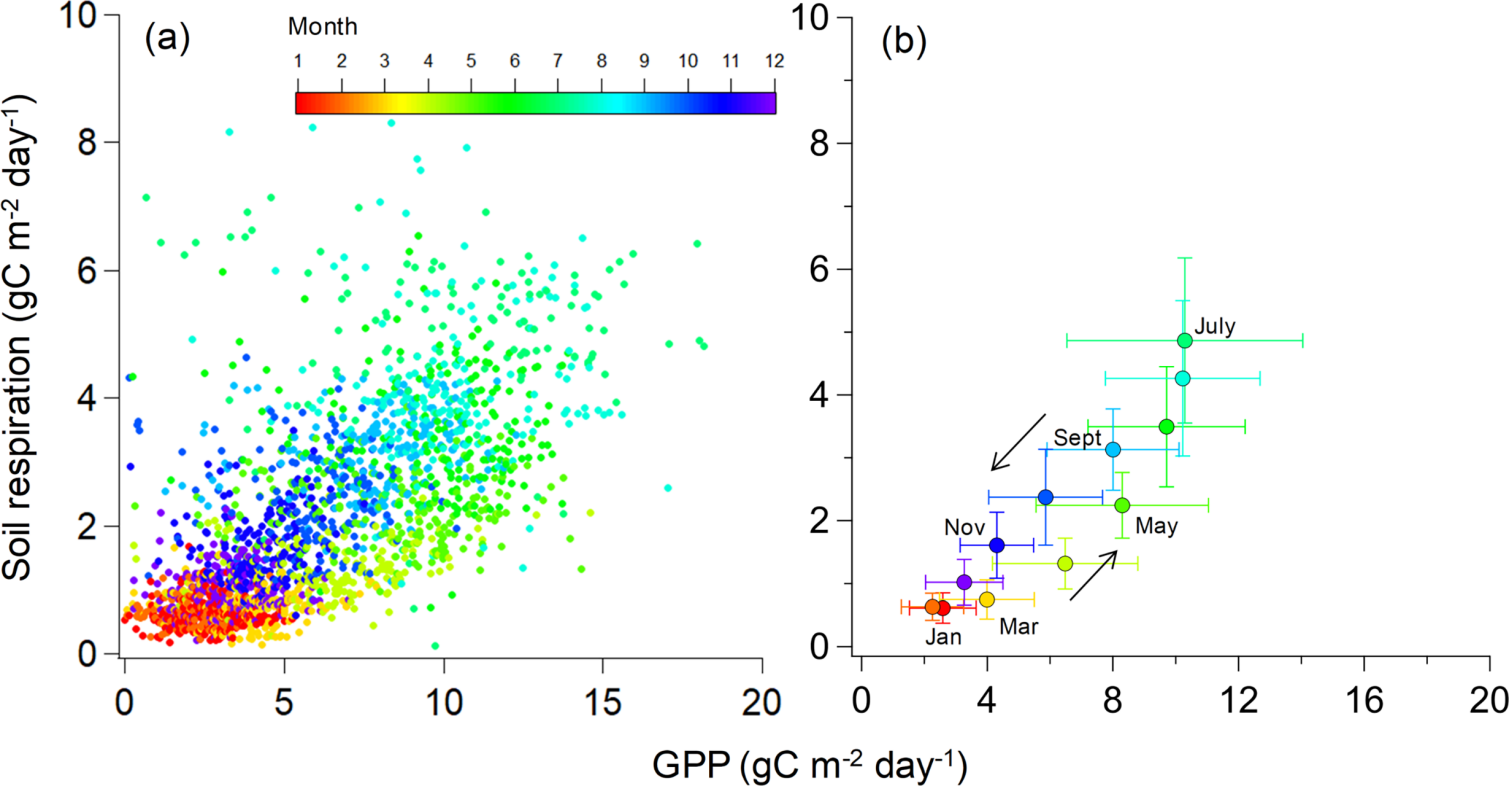
Relationship between daily soil respiration and gross primary production (GPP) during 2005–2010. (a) Each point represents an individual daily observation. (b) Each point is a mean value (± SD) for samples within a month. Color distributions were convergent in the monthly data.

There was a seasonal relationship between GPP and *R*_s_ of an evergreen conifer (Fig 3). We observed greater *R*_s_ relative to GPP in autumn for September to November when compared with spring for March to May.

### Seasonal patterns in *Q*_10_ and *E*_a_ values

**Fig 4:**
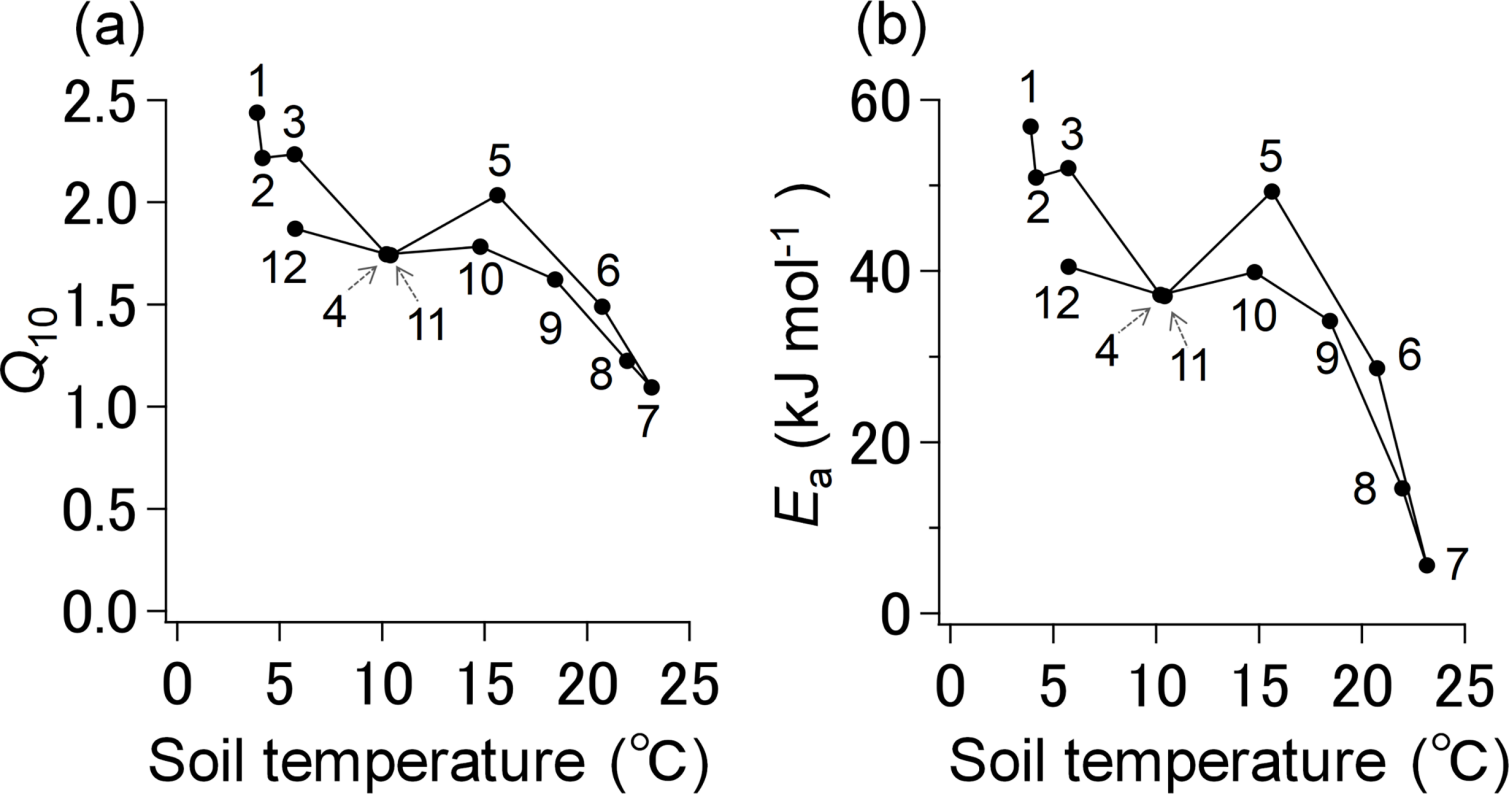
**Relationship between (a) *Q***_**10**_ **and (b) activation energy (*E***_**a**_**) of soil respiration and temperature for each month.** Numbers in the figure indicate months.

The *Q*_10_ and *E*_a_ values of the monthly *R*_s_ were 1.09–2.43 and 5.61–56.89 kJ mol^−1^, respectively (Table 2). Changes in *Q*_10_ and *E*_a_ values were related to seasonal patterns; the values were higher in winter than in summer. For all collected samples, the *Q*_10_ and *E*_a_ values of *R*_s_ declined markedly with increasing soil temperature, according to the seasons, which explained a significant proportion of the variation in the temperature sensitivity of *R*_s_ (*r* = 0.88, *p* < 0.001; Fig 4A, *r* = 0.83, *p* < 0.001; Fig 4B).

**Table 2:** Mean soil temperature, *Q*_10_, and activation energy (*E*_a_) for each month during 2005–2010.

Month	Soil temperature	*Q*_10_	*E*_a_
	° C		kJ mol^−1^
1	3.90	3.90	56885
2	4.16	2.08	50937
3	5.73	1.91	52017
4	10.21	2.55	37197
5	14.78	2.96	39873
6	18.44	3.07	34177
7	21.95	3.14	14599
8	23.15	2.89	5613
9	20.74	2.30	28634
10	15.62	1.56	49293
11	10.41	0.95	37084
12	5.75	0.48	40521

### Diurnal variation in soil respiration with seasons

**Fig 5:**
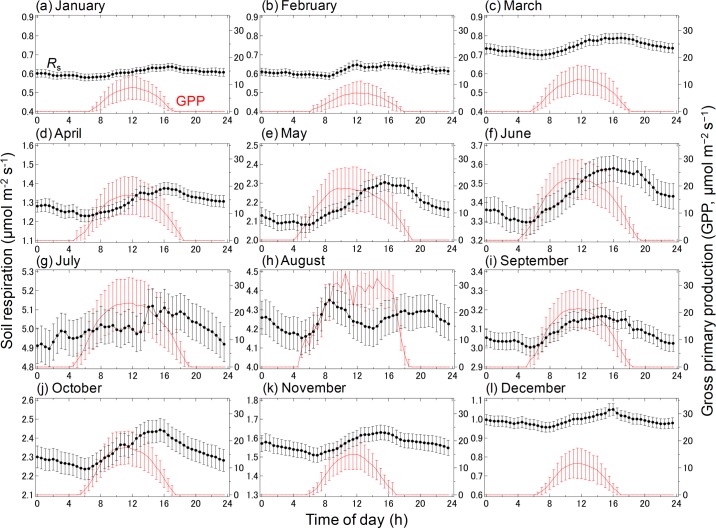
Diurnal variations in soil respiration and gross primary production (GPP) for each month. Error bars represent the standard errors of the mean for each month from 2005 to 2010. Each figure shows the fixed-width from bottom to top in Y-axis in all months.

Fig 5 shows the monthly time course of *R*_s_ and GPP. On a diurnal scale, *R*_s_ rates were frequently higher from 1200 to 1800 h, decreasing overnight and reaching their minimum values in the early morning. GPP was highest at 1100–1300h and decreased slightly during the afternoon. There was a lag between the time when maximum GPP and maximum *R*_s_ were reached.

**Fig 6:**
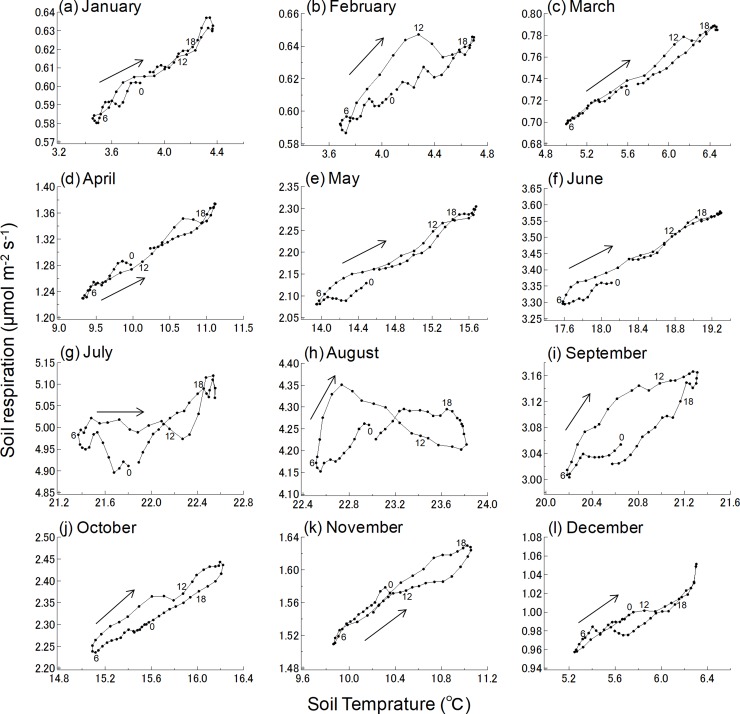
Relationship between soil respiration and temperature for each month. Each point indicates the mean value. Numbers in the figure indicate time of day of the mean for each month from 2005–2010.

A relationship between diurnal *R*_s_ and soil temperature was observed for each month, and a strong seasonal fluctuation in the relationship was also observed (Fig 6). For example, the diurnal pattern of *R*_s_ rates during July and August differed from that in other seasons. In August after the morning peaks, the *R*_s_ rates decreased around noon but soil temperatures remained high. *R*_s_ recovered in the afternoon, lagging behind the peak in soil temperature and resulting in a figure-eight curve (Fig 6H). In September and October, *R*_s_ relative to the temperature was higher in the morning than in the night, despite nearly constant soil temperatures (Fig 6I and 6J). Therefore, diurnal *R*_s_ rates showed a hysteresis pattern in seasons with high biological activity (Fig 6). In contrast, the *R*_s_ rates in seasons where biological activity ceases changed exponentially and showed negligible hysteresis.

## Discussion

From six years of observation by automated chambers, we characterised the magnitude and pattern of seasonal and diurnal *R*_s_ in an evergreen coniferous forest. This information may enable more accurate prediction of soil C dynamics and their associated ecosystem processes.

Our results support the hypothesis that high-frequency observations of *R*_s_ rates clearly indicate the seasonal changes in the response of *R*_s_ to soil temperature in field conditions, so that soil temperature alone is clearly insufficient to predict *R*_s_. In this study, *R*_s_ increased exponentially with increasing soil temperature. This correlation explained 80% of the variation in *R*_s_ across seasons when the best-fit Arrhenius model was used. In addition, the temperature sensitivity in this study was consistent with the findings of previous studies [45]. Our *Q*_10_ values were well within the global median of 2.4 [46] and the range (2.0–6.3) reported for European and North American forest ecosystems [47,48]. The Arrhenius function reveals the reactions with *E*_a_ around 50 kJ mol^−1^ [7], in agreement with our field observations. Nevertheless, there was a strong seasonal fluctuation in the relationship between *R*_s_ and soil temperature. The predicted *R*_s_ underestimated the actual *R*_s_ for June and July and overestimated *R*_s_ for August and September (Fig 2). Our results corroborate those of previous studies that reported increases in the contributions of *R*_s_ to ecosystem respiration during early summer [14,49]. This is probably due to the compensation of the model bias in late summer and autumn (overestimation) and early summer (underestimation), without explicit dependence of *R*_s_ on phenological attributes.

We found that there was a hysteresis in the seasonal relationship between GPP and *R*_s_ of an evergreen conifer (Fig 3). Seasonal patterns in *R*_s_ rates may be due to root production and respiration levels. Endogenous and phenological C assimilation rates are strongly correlated with belowground C allocation to roots, mycorrhizae, and rhizosphere microorganisms [28,29,50,51]. Root growth is assumed to peak early in the growing season and is therefore correlated with aboveground growth [52]. When a pulse of root growth occurs to support leaf production, the amount of respiring tissue and root CO_2_ emission simultaneously increase. In this study site, GPP relative to the solar radiation and temperature was higher during the spring and summer [40]. Kosugi et al. [40] noted that red leaf pigmentation in the winter prevented light inhibition at low temperatures and affected stomatal conductance and photosynthetic rates in an evergreen coniferous forest. Substrate limitation in the rhizosphere during the winter may reduce root growth and autotrophic respiration rates. Therefore, seasonal plant phenology patterns may lead to variation in the substrate supply and belowground C allocation, and partly affect variation in *R*_s_ [39].

The level of heterotrophic respiration is also indicative of the seasonal patterns of *R*_s_, particularly for the decline in observed *R*_s_ rates during August and September. In Asian monsoon areas, microbial decomposition is often enhanced during the early summer rainy season and suppressed by the late summer drought conditions [53]. Heterotrophic respiration is sensitive to seasonal rainfall patterns because soil water content strongly affects microbial physiology [12]. The biodiversity and metabolic activity of most soil microbial communities decrease with soil water content [54,55]. In fact, we found a significantly negative relationship between the temperature sensitivity of *R*_s_ and temperature; monthly *Q*_10_ and *E*_a_ were highest in winter and lowest in summer (Fig 4). These seasonal patterns in temperature sensitivity may be related to degradation of soil C, microbial physiological acclimation and community adjustment [55,56] by changing their lipid composition, synthesizing new proteins, and changing resource allocation from growth to survival mechanisms [57,58]. Previous studies reported that heterotrophic respiration and nutrient mineralization under drought also declined [58–60]. Consequently, the decline in *R*_s_ during the late summer is mostly related to a changed temperature response due to changed sensitivity of microbial degradation to water stress.

However, the seasonal *R*_s_ pattern in the present study contrasts with those reported previously [61]. Lee et al. [62] showed that *R*_s_ in a cool-temperate Japanese deciduous broad-leaved forest was lower in spring and early summer than in late summer and autumn. This difference may be explained by seasonal changes in soil heat transport and CO_2_ fluxes [34,63]. In spring, when soils are covered with snow, the contributions of root and microbial activity are reduced by the low temperatures in deeper soil layers, but the opposite occurs in late summer and autumn. In late summer, the *R*_s_ components increase in response to the warming of the deeper soil layers. Soils usually warm from the top downward in spring and cool from the top downward in autumn. The presence of snow and the timing of early spring thaw and late autumn frost affect the vertical distribution of soil temperature. In addition, high *R*_s_ in a deciduous forest in autumn could also be related to the high input of litter during autumn. Therefore, variation in CO_2_ production with soil depth during the growing season may affect heat transport-based hysteresis.

The coordination of aboveground and belowground phenological patterns would contribute to the seasonality of the *R*_s_ diurnal scale hysteresis. In September and October, *R*_s_ relative to the soil temperature was higher in the morning than at night. Diurnal hysteresis in the relationship between *R*_s_ and soil temperature is an example of multiple processes interacting to produce highly variable photosynthetic attributes [30,31]. Liu et al. [17] showed that the diurnal cycle of *R*_s_ in a mixed deciduous forest was related more to differences in photosynthetically active radiation than to variations in soil environmental conditions, suggesting that diurnal *R*_s_ patterns were associated with photosynthesis. In the present study, diurnal *R*_s_ was higher in the morning than in the nighttime, especially in September and October. The diurnal *R*_s_ pattern of the relationship between *R*_s_ and soil temperature showed a hysteresis loop. The *R*_s_ morning peaks in September and October suggest faster transfer of recent photosynthates to belowground in warm-temperate ecosystems. In fact, the *R*_s_ peaks occurred later than GPP peaks (Fig 5I and 5J). Our results suggest that soil temperature does not fully explain variations in diurnal *R*_s_ dynamics.

Interestingly, figure-eight-shaped diurnal *R*_s_ patterns were observed in July and August (Fig 6). This finding suggests that midday declines in root physiological activity may have occurred in early summer. Under natural field conditions, plants adapt to changes in the prevailing irradiance to protect and optimize photosynthesis. As a result, continuous daily variations occur. Photooxidative damage to leaf thylakoid membranes causes photoinhibition and stomatal closure. The leaf protects the photosynthetic apparatus by down-regulating it at higher temperatures under high photon flux [64]. Photoinhibitory damage and stomatal closure contribute significantly to midday photosynthetic depression and, indirectly, to the decline in C supply to the root system. Makita et al. [31] showed that weather conditions under high temperature stress cause a midday depression of CO_2_ assimilation in deciduous trees and then a sharp reduction in autotrophic respiration rate. The flux of new photosynthate to the rhizosphere significantly accelerates microbial activity there. This process affects the relative amount of heterotrophic respiration from decomposition of soil organic matter [33,65]. The results of the present study indicate how canopy processes affect the phase lags between the diurnal signals of soil temperature and forest floor *R*_s_. Some studies have suggested that the autotrophic component of *R*_s_ is controlled by carbohydrate production and internal transport in trees more than by diurnal variations in environmental variables [13,30]. Therefore, diurnal variation in *R*_s_ may explain the hysteresis loop observed in this study. Nevertheless, there remains some debate over the relative importance of temperature- and substrate-dependent processes as drivers of midday photosynthesis depression in actual *R*_s_ rates. There is little evidence that root growth and other C sinks are determined by substrate availability [66]. The associations between photosynthesis and *R*_s_ may be controlled by multiple factors, including photosynthate transport distance, root depth, plant physiology, growth stage, and environmental conditions [15,67]. Recent advances in isotopic labeling techniques have enabled the quantification of C partitioning in forests and the assessment of its role in tree growth, resource acquisition, and C sequestration at temporal scales [37,38]. Further investigation is needed to establish the mechanisms of aboveground–belowground interactions and the factors that control them.

In conclusion, continuous monitoring of *R*_s_ rates in a warm-temperate evergreen coniferous forest with an automated chamber system demonstrated diverse biological phases of the *R*_s_ rate at different time scales independently of soil temperature. We found that the magnitude and pattern of temporal *R*_s_ was depend on seasons across the temperature dependence. Additionally, more research is needed to elucidate whether the impact of linkage between aboveground and belowground C allocation depends on vegetation types and features of the soil environment, such as moisture. Soil CO_2_ efflux data with a high temporal resolution would help to quantify the contributions of abiotic and biotic effects on C flux and sequestration in forest soils.

## Supporting information

S1 Fig Mean monthly air temperature (°C) precipitation (mm) for the period 2005–2010. Error bars represent standard diviations. Data were from Y. Kosugi et al. [40].
(TIF)
