# Supplementary figures and images for: Seasonal and diurnal patterns of soil respiration in an evergreen coniferous forest: Evidence from six years of observation with automatic chambers
Source: PLoS One. 2018 Feb 12;13(2):e0192622. doi: 10.1371/journal.pone.0192622 (PMC5809067; doi:10.1371/journal.pone.0192622)

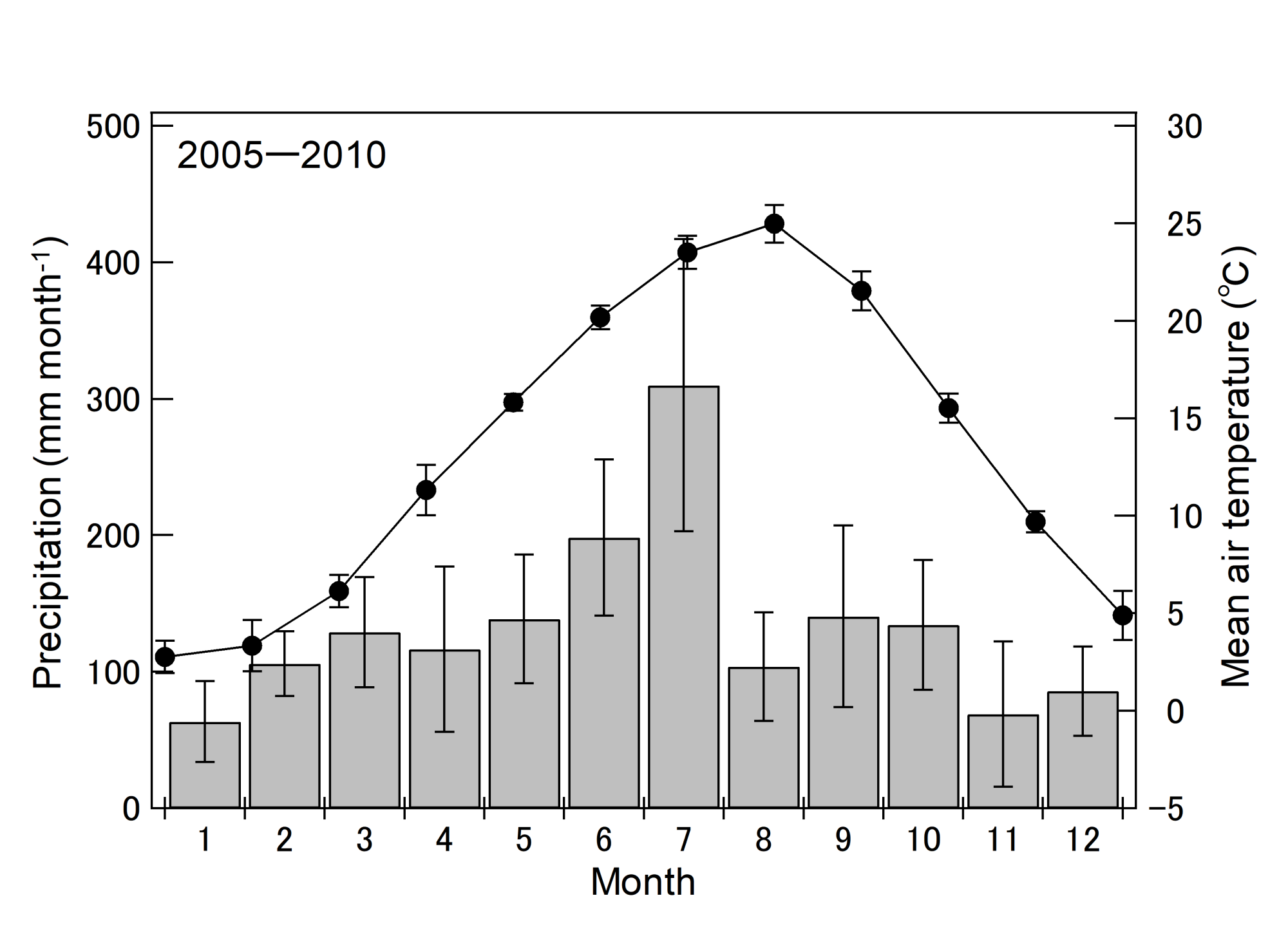

Supplement: S1 Fig — Error bars represent standard diviations. Data were from Y. Kosugi et al. [40]. (TIF) [file pone.0192622.s001.tif]
